# Generalized *GW*+Boltzmann Approach for the Description of Ultrafast Electron Dynamics in Topological Insulators

**DOI:** 10.3390/ma10070810

**Published:** 2017-07-17

**Authors:** Marco Battiato, Irene Aguilera, Jaime Sánchez-Barriga

**Affiliations:** 1 Institute of Solid State Physics, Vienna University of Technology, A-1040 Vienna, Austria; battiato@ifp.tuwien.ac.at; 2 Peter Grünberg Institute and Institute for Advanced Simulation, Forschungszentrum Jülich and JARA, D-52425 Jülich, Germany; i.aguilera@fz-juelich.de; 3 Helmholtz-Zentrum Berlin für Materialien und Energie, Albert-Einstein-Str. 15, 12489 Berlin, Germany

**Keywords:** topological insulators, ultrafast dynamics, many-body perturbation theory, Boltzmann approach, time- and angle-resolved photoemission spectroscopy

## Abstract

Quantum-phase transitions between trivial insulators and topological insulators differ from ordinary metal-insulator transitions in that they arise from the inversion of the bulk band structure due to strong spin–orbit coupling. Such topological phase transitions are unique in nature as they lead to the emergence of topological surface states which are characterized by a peculiar spin texture that is believed to play a central role in the generation and manipulation of dissipationless surface spin currents on ultrafast timescales. Here, we provide a generalized GW+Boltzmann approach for the description of ultrafast dynamics in topological insulators driven by electron–electron and electron–phonon scatterings. Taking the prototypical insulator Bi${}_{2}$Te${}_{3}$ as an example, we test the robustness of our approach by comparing the theoretical prediction to results of time- and angle-resolved photoemission experiments. From this comparison, we are able to demonstrate the crucial role of the excited spin texture in the subpicosecond relaxation of transient electrons, as well as to accurately obtain the magnitude and strength of electron–electron and electron–phonon couplings. Our approach could be used as a generalized theory for three-dimensional topological insulators in the bulk-conducting transport regime, paving the way for the realization of a unified theory of ultrafast dynamics in topological materials.

## 1. Introduction

A huge range of technologically relevant properties of materials rely on the shape of the density of states within a narrow energy range around the Fermi energy [1]. The singly most important parameters are the amplitude (or the absence) of a band gap and its position with respect to the Fermi energy. These critically impact properties ranging from electrical conductivity to the absorption and reflectivity of materials. It is therefore clear why increasing effort is being devoted to the control of materials’ band gap [2]. Metal-to-insulator transitions allow for dramatically tuning the properties of a material, and have a profound technological significance [3].

The control of a metal-to-insulator transition is usually achieved by doping. However, recently, a fascinating technique has been developed of inducing dynamic doping through femtosecond laser excitation [4,5,6,7]. Topological insulators provide a further playground to study this transition, since their properties naturally show a position-dependent transition from an insulating regime in the bulk to a metallic one on the surface [8]. This position-dependent metal-to-insulator transition can also be coupled to the already mentioned and more standard techniques of static or dynamic doping, allowing for unprecedented control.

Differently from ordinary metal-to-insulator transitions, topological quantum-phase transitions proceed through a transition region where the bulk band gap closes and re-opens, and the resulting topological phase respects the fundamental principle of bulk-boundary correspondence [9]. Moreover, due to a band inversion in the bulk caused by strong spin–orbit coupling, the topological side of the quantum-phase transition hosts topological surface states with unique properties [9,10,11], and these do not exist in ordinary metal-to-insulator transitions. Topological surface states are characterized by a peculiar spin texture where electron spins are locked to their linear momentum [12,13,14,15]. This spin texture is believed to play a central role in the generation and manipulation of dissipationless surface spin currents on ultrafast timescales [16,17,18,19], as well as in realizing exotic magnetic-spin physics [20] and future spin-based low power transistors and devices.

The use of femtosecond laser pulses also offers a unique alternative to achieve efficient control of topological spin currents on subpicosecond time scales. In particular, ultrafast dynamics in topological insulators opens the route to new concepts of devices of superior performance, as it would allow increasing the speed of information transport up to frequencies a thousand times faster than in modern electronics. Recent experiments using time- and angle-resolved photoemission (tr-ARPES) have revealed the importance of bulk-mediated scattering in the decay process of hot electrons across the linear energy-momentum dispersion of topological surface states [21,22,23,24,25,26,27,28]. Similar experiments also allowed the observation of Floquet–Bloch excitations [29], which might open the door to the generation of a transient-anomalous quantum Hall effect on ultrafast time scales [30] without the need of magnetic dopants or applied magnetic fields [31].

However, to be able to tackle these new fascinating opportunities of the topological quantum-phase transitions in these materials, material-specific theoretical understanding and modelling are fundamental. Equilibrium descriptions are insufficient to address the problem of the picosecond and subpicosecond dynamics, and even more critically the process of dynamical doping. To this aim, we develop here a theoretical approach for the description of the ultrafast dynamics in topological insulators by combining two very successful methods, the Boltzmann approach and the GW approximation. While the Boltzmann approach has been proven successful for the description of dynamics of materials [32,33,34,35], an improvement on the basis of state-of-the-art many-body calculations including all possible scattering effects is mandatory. GW is one of the most accurate theoretical approaches for the description of band structures [36,37,38,39,40], and up to date, a generalized GW+Boltzmann formulation has not been developed.

Therefore, in the present work, we use GW band structure calculations to compute within the Boltzmann approach the dynamics of excited electrons in topological materials. Taking the prototypical topological insulator Bi${}_{2}$Te${}_{3}$ as an example, our approach is proven to have a manageable computational cost while retaining a remarkable accuracy when compared to experiments, and especially the ability of predicting and describing complex, non-trivial out-of-equilibrium dynamics.

The manuscript is organized as follows: In Section 2.1 and Section 2.2, we introduce the theoretical and experimental methods. In Section 3.1, we derive the GW+Boltzmann approach by including the effect of electron–electron and electron–phonon scatterings explicitly. In Section 3.2 and Section 3.3, we compare the theoretical and experimental results. Finally, a summary and outlook is given in Section 4.

## 2. Methods

### 2.1. Theory

The GW calculation and its starting point within density functional theory (DFT) were carried out with the all-electron full-potential linearized augmented-plane-wave (FLAPW) codes spex [41] and fleur [42], respectively. The FLAPW method allows for a treatement on an equal footing of core, valence, and conduction electrons. The DFT electronic density was determined employing the local-density approximation (LDA) for the exchange-correlation functional. To include relativistic effects in the calculations, the core electrons were treated fully relativistically by solving the Dirac equation. For the valence electrons, space is partitioned into an interstitial region, where relativistic effects are neglected, and atomic spheres, where the scalar-relativistic approximation is used and the spin–orbit coupling is incorporated self-consistently employing the “second variation” technique [43]. In the GW calculations, we take the spin–orbit coupling into account by using an explicit spin-dependent formalism [44,45], in which the self-energy acquires spin off-diagonal blocks. Thus, the spin–orbit coupling in included in the calculation of the Green function *G*, of the screened Coulomb interaction *W*, and, therefore, of the self-energy.

In the atomic spheres, we used an angular momentum cutoff $l_{\max}=10$ and in the interstitial region a plane-wave cutoff of 4.5 ${\mathrm{bohr}}^{-1}$. A mixed product basis [41,46] was used to represent the two-particle quantities in the GW calculation, such as the dielectric matrix and the screened interaction. This basis was constructed with an angular momentum cutoff of l=5 and a linear momentum cutoff of 2.9 ${\mathrm{bohr}}^{-1}$. The screened interaction is calculated within the random-phase approximation without resorting to a plasmon-pole model for the frequency dependence. The frequency convolution of the self-energy is evaluated with the use of a contour integration technique on the complex frequency plane [47,48]. We go beyond the widely used *perturbative* solution of the quasiparticle equation by taking into account off-diagonal elements of the self-energy [49]. To compute the Green function and the polarization function, 500 bands had to be included in order to obtain the wanted accuracy. An 8 × 8 × 8 **k**-point grid was used to sample the Brillouin Zone. Local orbitals were used to describe the semicore *d*-states of Bi and Te, as well as high-lying states to avoid linearization errors [50,51]. We used the experimental lattice structure of [52].

Due to the high computational demands of the GW method, calculations of thick films (where bulk and surface states and surface resonances coexist) would be extremely costly. Therefore, in this work, we construct tight-binding Hamiltonians for the description of the surface states in a film geometry. The corresponding tight-binding parameters are obtained ab initio from the GW calculations of the bulk, with the help of Wannier functions [53]. This method has been previously used for this family of materials (see e.g., Ref. [54]), but the tight-binding parameters were always obtained from DFT calculations.

The time-dependent photoemission calculations were performed within the Boltzmann approach [55,56,57]. The dynamical calculations take into account all possible spin-dependent electron transitions in energy-momentum space as well as electron–electron and electron–phonon scatterings in a quantitative way (for more details, please see Section 3.1).

### 2.2. Experiment

Experiments were performed at room temperature under ultrahigh vacuum conditions with a base pressure below $1\;\times \;10^{-10}$ mbar. Photoelectrons were detected with a Scienta R8000 electron analyzer (Scienta Omicron, Uppsala, Sweden) and the base pressure of the experimental setup was better than $1\;\times \;10^{-10}$ mbar. Laser-based tr-ARPES experiments were performed using pump (1.5 eV) and probe (6 eV) pulses from a home-made fs Ti:Sapphire oscillator coupled to an ultrafast amplifier laser system (RegA, Coherent, Dieburg, Germany). The repetition rate of the laser was 150 kHz, and the time resolution of the tr-ARPES experiment was ∼180 fs. The angular and energy resolutions of the measurements were $0.1^{\circ }$ and 20 meV, respectively.

To generate the fourth harmonic of the laser, we employed three steps of sum-frequency generation processes. In a first step, the output laser beam with central wavelength of 800 nm was focused into a thin barium borate (BBO) crystal to produce the second harmonic (3 eV) via type I phase-matching. Subsequently, the fundamental radiation and the 3 eV beam were mixed in a second BBO crystal producing the third harmonic (4.5 eV) via type II phase matching. In the last step, the third and the first harmonic were mixed (type I) to produce the fourth harmonic (6 eV). The resulting beams were spatially separated using a CaF${}_{2}$ prism. All the harmonics split by the prism were blocked except for the first and the fourth harmonic, which were focused on the sample. The pump fluence was ∼100 μJ/cm${}^{2}$, and the linear polarizations of the pump and probe beams were adjusted using zero-order tunable λ/2 waveplates. The pump-probe time delay Δt was varied using an optical delay stage allowing motorized linear translations of the mirrors with sub-μm resolution.

Bi${}_{2}$Te${}_{3}$ bulk single crystals were cleaved in situ and grown by the Bridgman method [58] at the Lomonosov Moscow State University. The growth temperature was in the range of 580–600 ${}^{\circ }$C, and the temperature gradient during growth was 5 ${}^{\circ }$C/cm. The high crystal quality of the obtained (111) surfaces was verified by low-energy electron diffraction as well as by the presence of sharp features in the ARPES dispersions.

## 3. Results and Discussion

### 3.1. Generalized Boltzmann Approach for Ultrafast Dynamics in Topological Insulators

To compute the dynamics of the momentum- and band-resolved electronic population at the surface, we used the Boltzmann equation after explicitly including electron–electron and electron–phonon scatterings in a quantitative way. The change of the population $f_{n}\left(k,t\right)$ in the *n*-th band and with momentum k was obtained by computing all the incoming and outgoing scatterings, yielding the expression:

(1) $$\begin{array}{c} \frac{\partial f_{n}\left(k,t\right)}{\partial t}=\\ -\sum_{n_{1},n_{2},n_{3}}\int W_{e-e}\left(n,k,n_{1},k_{1}\mapsto n_{2},k_{2},n_{3},k_{3}\right)\;f_{n}\left(k,t\right)f_{n_{1}}\left(k_{1},t\right)\left(1-f_{n_{2}}\left(k_{2},t\right)\right)\left(1-f_{n_{3}}\left(k_{3},t\right)\right)\;\cdot \\ \cdot dk_{1}dk_{2}dk_{3}+\sum_{n_{1},n_{2},n_{3}}\int W_{e-e}\left(n_{2},k_{2},n_{3},k_{3}\mapsto n,k,n_{1},k_{1}\right)\left(1-f_{n}\left(k,t\right)\right)\left(1-f_{n_{1}}\left(k_{1},t\right)\right)f_{n_{2}}\left(k_{2},t\right)\;\cdot \\ \cdot f_{n_{3}}\left(k_{3},t\right)dk_{1}dk_{2}dk_{3}-\sum_{n_{1}}\int (W_{e-\mathrm{ph}}\left(n,k,q\mapsto n_{1},k_{1}\right)g_{ph}\left(q,t\right)+W_{e-\mathrm{ph}}\left(n,k\mapsto n_{1},k_{1},q\right)(1+\;\\ +g_{ph}\left(q,t\right)))f_{n}\left(k,t\right)\left(1-f_{n_{1}}\left(k_{1},t\right)\right)dqdk_{1}+\sum_{n_{1}}\int (W_{e-\mathrm{ph}}\left(n_{1},k_{1},q\mapsto n,k\right)g_{ph}\left(q,t\right)+\;\\ +W_{e-\mathrm{ph}}\left(n_{1},k_{1}\mapsto n,k,q\right)\left(1+g_{ph}\left(q,t\right)\right))\left(1-f_{n}\left(k,t\right)\right)f_{n_{1}}\left(k_{1},t\right)dqdk_{1}+{\left.\frac{\partial f_{n}\left(k,t\right)}{\partial t}\right|}_{l}, \end{array}$$

where the first integral describes the decrease in population due to all the scatterings of electrons in the *n*-th band and momentum k with any other electron in band $n_{1}$ and momentum $k_{1}$, and arriving at any two states at bands $n_{2}$ and $n_{3}$ and momenta $k_{2}$ and $k_{3}$, respectively. The second integral represents all the combinations of scattering in other states that instead lead to an increase of population in the *n*-th band and momentum k. The next two integrals represent analogous scatterings involving one electron and one phonon. The last term represents the laser excitation.

We first focus on electron–electron scatterings. The scattering amplitude $W_{e-e}\left(k,k_{1}\mapsto k_{2},k_{3}\right)$ (where we dropped the explicit dependence on the band index for shortness) has to satisfy energy and momentum conservation, and can be written as

(2) $$\begin{array}{c} W_{e-e}\left(k,k_{1}\mapsto k_{2},k_{3}\right)=\omega_{e-e}\left(k,k_{1}\mapsto k_{2},k_{3}\right)\;\cdot \\ \cdot \delta \left(k+k_{1}-k_{2}-k_{3}\right)\;\delta \left(E\left(k\right)+E\left(k_{1}\right)-E\left(k_{2}\right)-E\left(k_{3}\right)\right), \end{array}$$

where E(k) is the two-dimensional band dispersion (again with the band index dropped for shortness) and the unconstrained scattering amplitude $\omega_{e-e}\left(k,k_{1}\mapsto k_{2},k_{3}\right)$ is a smooth function of the variables. The first Dirac delta enforces explicitly the momentum conservation, while the second one the energy conservation. We have ignored umklapp scatterings since, in the materials under investigation, the most relevant scatterings happen close enough to the Dirac point.

To reduce the complexity of the problem, we make the assumption that the two-dimensional surface band structure is circularly symmetric. This is a very good approximation close to the vertex of the Dirac cone. Within this approximation, the electron population f(k) depends only on the length *k* of the momentum k. Moreover, the integrals in Equation (1) can be partially calculated analytically. For instance, the first term in Equation (1) can be reduced to

(3) $$\begin{array}{c} \frac{\partial f(k,t)}{\partial t}=-\int dk_{1}dk_{2}dk_{3}\;P_{e-e}\left(k,k_{1}\mapsto k_{2},k_{3}\right)\cdot \\ \;\;\;\cdot \;f\left(k,t\right)f\left(k_{1},t\right)\left(1-f\left(k_{2},t\right)\right)\left(1-f\left(k_{3},t\right)\right), \end{array}$$

where, again, for shortness, we have dropped both the band index and the summations over the different bands. Computing the effective scattering probability P is a non trivial geometrical problem due to the momentum conservation.

**Figure 1:**
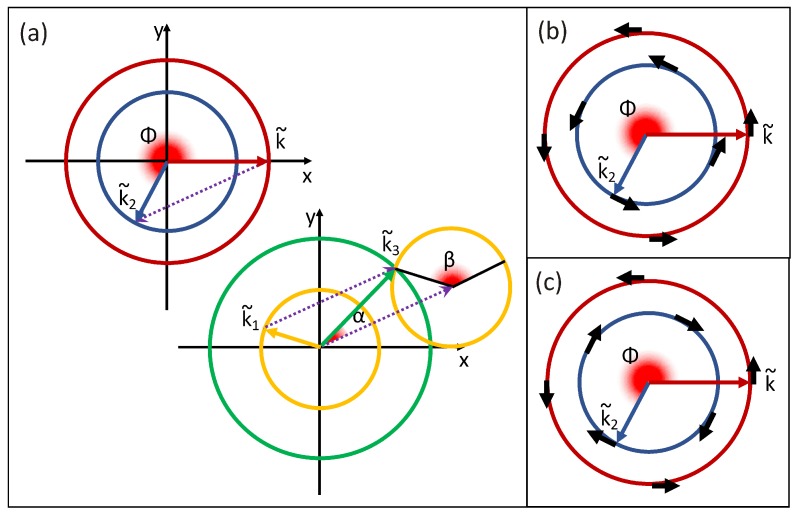
(**a**) geometrical representation of the momentum conservation for electron–electron scattering; (**b**,**c**) mutual orientation of the spin in initial and final states in case the two states have the same or opposite handedness, respectively.

We generalize here the approach to a general shape of the unconstrained scattering amplitude $\omega_{e-e}\left(k,k_{1},k_{2},k_{3}\right)$ as in Equation (2). We will however assume that $\omega_{e-e}\left(k,k_{1},k_{2},k_{3}\right)$ can be written as a function $\omega_{e-e}\left(k,k_{1},k_{2},k_{3},\phi ,\alpha -\beta \right)$ of the lengths *k*, $k_{1}$, $k_{2}$ and $k_{3}$, of the momenta k, $k_{1}$, $k_{2}$ and $k_{3}$, and of the angle ϕ between k and $k_{2}$, and the angle α−β between $k_{1}$ and $k_{3}$ (see Figure 1).

In that case, we obtain

(4) $$\begin{array}{c} P\left(k,k_{1},k_{2},k_{3}\right)=\delta \left(E\left(k\right)+E\left(k_{1}\right)-E\left(k_{2}\right)-E\left(k_{3}\right)\right)\;\cdot \\ \;\cdot \int_{\phi_{\min}}^{\phi_{\max}}\;\;\frac{16\;k_{1}\;k_{2}\;k_{3}\;\omega_{e-e}\left(k,k_{1},k_{2},k_{3},\phi ,\alpha -\beta \right)\;\;d\phi }{\sqrt{2k_{1}^{2}\;k_{3}^{2}+2\delta k^{2}\left(k_{1}^{2}+k_{3}^{2}\right)-\delta k^{4}-k_{1}^{4}-k_{3}^{4}}}, \end{array}$$

where, within the integral, the following expressions are to be used:

(5) $$\begin{array}{c} \delta k=\sqrt{k^{2}+k_{2}^{2}-2kk_{2}\cos\phi }, \end{array}$$

(6) $$\begin{array}{c} \phi_{\min}=\max\left[0,\arccos\left(\frac{k^{2}+k_{2}^{2}-{\left(k_{3}-k_{1}\right)}^{2}}{2kk_{2}}\right)\right], \end{array}$$

(7) $$\begin{array}{c} \phi_{\max}=\min\left[\pi ,\arccos\left(\frac{k^{2}+k_{2}^{2}-{\left(k_{3}+k_{1}\right)}^{2}}{2kk_{2}}\right)\right], \end{array}$$

(8) $$\begin{array}{c} \tilde{k}=k\;u_{x} \end{array}$$

(9) $$\begin{array}{c} {\tilde{k}}_{1}=k_{1}\left(\cos\left(\beta -\theta \right)\;u_{x}+\sin\left(\beta -\theta \right)\;u_{y}\right), \end{array}$$

(10) $$\begin{array}{c} {\tilde{k}}_{2}=k_{2}\left(\cos\left(\phi \right)\;u_{x}+\sin\left(\phi \right)\;u_{y}\right), \end{array}$$

(11) $$\begin{array}{c} {\tilde{k}}_{3}=k_{3}\left(\cos\left(-\alpha -\theta \right)\;u_{x}+\sin\left(-\alpha -\theta \right)\;u_{y}\right), \end{array}$$

(12) $$\begin{array}{c} \theta =\arccos\left(-\frac{k_{2}^{2}-k^{2}-\delta k^{2}}{2\;k\;\delta k}\right), \end{array}$$

(13) $$\begin{array}{c} \alpha =\arctan\;\left(\;\frac{\sqrt{2\delta k^{2}\left(k_{1}^{2}+k_{3}^{2}\right)-{\left(k_{1}^{2}-k_{3}^{2}\right)}^{2}-\delta k^{4}}}{k_{3}^{2}-k_{1}^{2}+\delta k^{2}}\right), \end{array}$$

(14) $$\begin{array}{c} \beta =\pi -\arccos\left(\frac{k_{1}^{2}+\delta k^{2}-k_{3}^{2}}{2\;k_{1}\;\delta k}\right). \end{array}$$

The unconstrained scattering amplitude $\omega_{e-e}\left(k,k_{1}\mapsto k_{2},k_{3}\right)$, in general, depends on the details of the wavefunctions of all the states involved. We use here a simplified shape which takes into account the spin direction of the four involved states. We assume that the two-dimensional states have spin-momentum locking (consistent with the assumption of circular symmetry), and either left- or right-handedness. We suppose that the unconstrained scattering amplitude $\omega_{e-e}\left(k,k_{1},k_{2},k_{3},\phi ,\alpha -\beta \right)$ is the product of a constant and two functions that provide a simplified estimation of the spin overlap of the initial and final state independently for both electrons involved in the transition. We write

(15) $$\begin{array}{cc} \omega_{e-e}\left(k,k_{1}\mapsto k_{2},k_{3}\right)\approx & \;\;W_{e-e}\;\frac{1+\cos(∡\left[\sigma_{k},\sigma_{k_{2}}\right])}{2}\cdot \\ & \cdot \frac{1+\cos(∡\left[\sigma_{k_{1}},\sigma_{k_{3}}\right])}{2}, \end{array}$$

where $W_{e-e}$ is a constant representing the absolute amplitude of the electron–electron scattering probability, $\sigma_{k}$ is the direction of the spin moment at k, and ∡[] indicates the angle between the two vectors within the brackets. The angle $∡[\sigma_{k},\sigma_{k_{2}}]$ is either ϕ or π−ϕ in case the two bands *n* and $n_{2}$ have the same or opposite handedness respectively. Similarly, $∡[\sigma_{k_{1}},\sigma_{k_{3}}]$ is either α−β or π−α+β. Equation (4) can now be written explicitly. However, there is no analytical expression for the integral, which can anyhow be computed numerically. To ensure the energy conservation in Equation (4), we discretize the bands as a piecewise constant functions, with values only on a uniform grid in energy.

The electron–phonon scattering is treated in a similar way, and depends on the electron–phonon scattering constant $W_{e-\mathrm{ph}}$, as in Equation (15). However, the phonon system is not treated explicitly and is considered as a bath with a fixed temperature ($T_{\mathrm{ph}}=300\;K$). Moreover, given the number of phononic bands and impurity states, we do not require the momentum conservation, but only energy. Therefore, we simply substitute $g_{ph}\left(q,t\right)$ with a Bose–Einstein distribution for scatterings with energy transfer up to 0.15 eV.

Finally, the laser excitation is assumed to have a Gaussian shape in time:

(16) $$\begin{array}{c} {\left.\frac{\partial f_{n}\left(k,t\right)}{\partial t}\right|}_{l}=\exp^{\frac{t^{2}\ln16}{\delta t^{2}}}\cdot (\sum_{n_{1}}\\ \int S_{\mathrm{ext}}\left(n_{1},k_{1}\mapsto n,k\right)f_{n_{1}}\left(k_{1},t\right)\left(1-f_{n}\left(k,t\right)\right)dk_{1}+\\ -\int S_{\mathrm{ext}}\left(n,k_{1}\mapsto n_{1},k\right)f_{n}\left(k,t\right)\left(1-f_{n_{1}}\left(k_{1},t\right)\right)dk_{1}), \end{array}$$

where δt is the time width of the laser pulse. In spite of the fact that the laser excitation tends to have a narrow spectrum, we allow all excitations up to the maximum frequency (and with equal probability). This choice has been made because we will simplify the band structure, by not treating explicitly the states below the Dirac point, but by substituting those states with flat valence bands at the same energy. Therefore, to prevent an unrealistic pumping only at a single final energy, we have allowed the laser to excite a wide range of transitions.

### 3.2. GW Calculations in Equilibrium: Application to Bi${}_{2}$Te${}_{3}$

**Figure 2:**
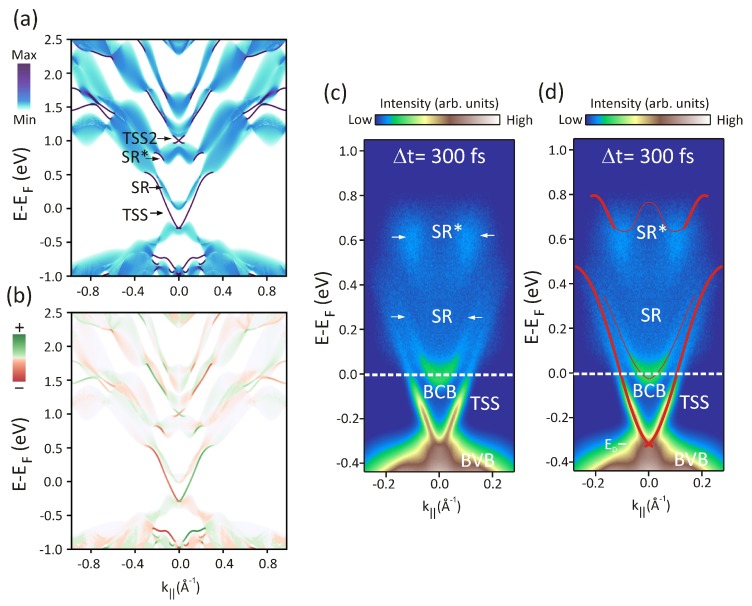
(**a**) band structure of a 100 quintuple layers slab of Bi${}_{2}$Te${}_{3}$ obtained with a tight-binding model based on GW. The color scheme represents the localization of the states on the topmost quintuple layer; (**b**) corresponding calculations of the spin polarization, obtained as the expectation value of the $\sigma_{y}$ Pauli matrix. The color map represents the magnitude and direction of the spin polarization component perpendicular to the electron momentum; (**c**) snapshot of the Bi${}_{2}$Te${}_{3}$ band structure measured 300 fs after laser excitation; (**d**) comparison to the GW calculated band dispersions, superimposed as red solid lines on the data shown in (**c**).

Taking the prototypical insulator Bi${}_{2}$Te${}_{3}$ as an example, in the following, we test the robustness of our approach by comparing the theoretical calculations to results of time- and angle-resolved photoemission experiments. Thus, in this section, we will firstly discuss the equilibrium electronic structure of Bi${}_{2}$Te${}_{3}$. Figure 2a shows a calculation of a slab of 100 quintuple layers of Bi${}_{2}$Te${}_{3}$ obtained with a tight-binding model based on GW. The color scheme represents the localization of the states on the topmost quintuple layer: Purple states are surface states, light blue states are bulk-like states, and some surface resonances are visible as dark blue features. The topological surface state (TSS), a higher-energy Dirac cone labeled TSS2, and two surface resonances labeled SR and SR* are clearly seen.

As discussed in the previous section, the ground-state spin orientation plays a key role in the dynamics. Therefore, we show in the color map of Figure 2b the calculated spin polarization, which is obtained as the expectation value of the $\sigma_{y}$ Pauli matrix. It should be noted that in GW calculations, it is common to neglect the off-diagonal elements of the self-energy. This perturbative approach corresponds to implicitly assuming that the LDA wave functions are good approximations to the quasiparticle ones. This would fix the spin polarization at the LDA level and would not allow it to change in the many-body calculation. This has been shown to be particularly inaccurate for Bi${}_{2}$Te${}_{3}$ [49], and, therefore, we solve the quasiparticle equation instead, obtaining the full self-energy matrix. With this method, the spin texture and the magnitude of the spin polarization can be calculated from the quasiparticle wave functions.

To directly compare the results of the theoretical calculations to the experiment, in Figure 2c, we show a snapshot of the band structure measured at a pump-probe delay of Δt=300 fs after laser excitation, and, in Figure 2d, we superimpose the calculated band dispersions (red color) on the experimental result shown in Figure 2c. Differently from conventional ARPES, which does not allow for accessing the band structure above the Fermi level, in Figure 2c, we clearly resolve the intensity contributions from the different states not only below but also above the Fermi level. In particular, due to the energy of the infrared pump pulse, we observe excitations up to E-E${}_{F}∼0.8\mathrm{eV}$. The experimental data show a gapless Dirac cone representing the TSS together with the intensity contributions from SR and SR* states, in agreement with the calculations. In addition, we observe distinct contributions from the bulk-conduction band (BCB) at the Fermi level and from the bulk-valence band (BVB) below the Dirac point (E${}_{D}$).

Interestingly, the SR states are similar to the ones previously observed in Bi${}_{2}$Se${}_{3}$ [59], and are located at the border of a surface-projected bulk band gap above the BCB top. The SR* states, on the other hand, do not yet have a known analog in Bi${}_{2}$Se${}_{3}$ and lie within a second projected bulk band gap at higher energies. According to our calculations, both SR and SR* states are topologically trivial, and thus it is remarkable to see that they possess a spin texture locked to the electron momentum and a relatively high spin polarization. The origin of their spin textures arises from band inversion, as it can be understood from the evolution of the system as it goes through a topological quantum-phase transition from a trivial insulator to a topological insulator [59]. In particular, both SR and SR* states arise from Rashba-split bands that persist on the trivial side of the topological quantum phase transition, and, therefore, they are completely different from unpolarized, topologically trivial, three-dimensional states that are usually responsible for conventional, non-topological, metal-to-insulator transitions in the bulk.

Overall, as seen in Figure 2c,d, we find very good agreement between calculations and experiment, not only concerning the energy positions of the different bands but also their energy-momentum dispersions. Nevertheless, some very small differences can still be observed between the calculated and measured electronic structures, particularly concerning the energy position of SR* states, which are slightly shifted to higher energy in the calculation. We attribute this to the neglect of excitonic effects in the GW calculations, which introduce slight deviations in the energy positions of the unoccupied bands. Since GW misses electron–hole interactions, a further improvement in the comparison between experiment and theory could be achieved, for instance, by the solution of the Bethe–Salpeter equation. This would allow for including excitonic effects at the level of many-body perturbation theory, which is an important task for future studies. While this approach would be at present too computationally demanding for this family of materials, its implementation in the framework of GW+Boltzmann calculations would represent an important step forward as it has not been done so far. In spite of being a real computational challenge, GW calculations are, to date, the most accurate calculations of the electronic structure of this family of materials.

### 3.3. Parameter-Sensitive GW+Boltzmann Approach for the Description of Subpicosecond Electron Dynamics in Bi${}_{2}$Te${}_{3}$

Having discussed the comparison between the results of GW calculations and experiments on the electronic structure of Bi${}_{2}$Te${}_{3}$, in this section, we will test the accuracy of the GW+Boltzmann approach developed in Section 3.1 by comparing to results of tr-ARPES experiments on this system. In particular, we will see that the spin-polarized SR and SR* states, which are born from a trivial-to-topological quantum phase transition together with the TSS, not only play a key role in the dynamics but also strongly influence the ultrafast decay of excited electrons within the TSS bands following laser excitation.

**Figure 3:**
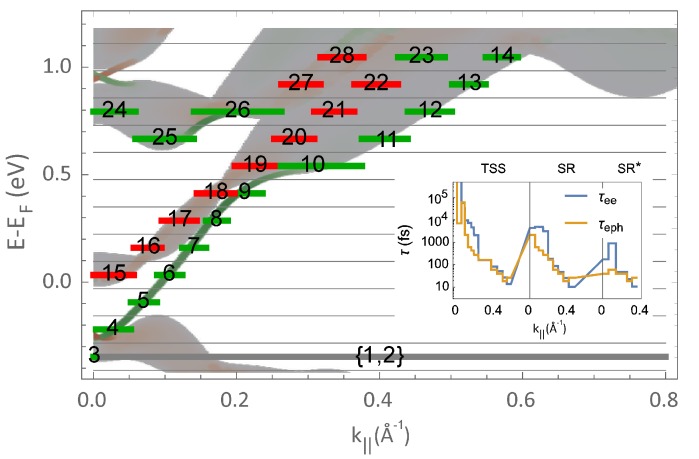
Electron bands used in the Boltzmann calculations compared to the GW band structure of Bi${}_{2}$Te${}_{3}$. The Boltzmann dynamics was solved numerically by discretizing the GW bands and their spin orientation. Red and green colors correspond to opposite spin states, and the spin orientations are locked to the electron momentum. Inset: electron–electron and electron–phonon scattering lifetimes, obtained by computing the *k*-dependent decay time of a small excitation above the room temperature Fermi–Dirac occupation. The horizontal axis is split along the electron wave vector parallel to the surface, and follows the calculated energy-momentum dispersions of individual bands, labeled TSS, SR and SR*.

Taking into account the GW calculated Bi${}_{2}$Te${}_{3}$ bands as an input, we solved the Boltzmann dynamics numerically and as a function of only two parameters, $W_{e-e}$ and $W_{e-ph}$. To do this, we firstly discretized the GW bands and their spin orientation as depicted in Figure 3, where red and green colors correspond to opposite spin states. Following the pump laser pulse, electrons excited into SR* states are transferred into the lower energy SR and TSS states; however, the scattering process is fully constrained by their alternating spin textures. Thus, our calculations reveal that both types of scatterings, electron–electron and electron–phonon, lead to completely different dynamical behaviors associated to the specific relaxation of the different bands. This allows the unambiguous estimation of the two parameters $W_{e-e}=0.0028$ nm${}^{3}$/fs and $W_{e-ph}=0.1$ nm/fs. With these values, we can immediately evaluate the electron–electron and electron–phonon scattering lifetimes at room temperature, as shown in the inset of Figure 3. This is done by computing the *k*-dependent decay time of a small excitation above the room temperature Fermi–Dirac occupation. Specifically, the inset displays band-resolved scattering lifetimes, where the horizontal axis spans along the electron wave vector parallel to the surface following the calculated energy-momentum dispersions of individual bands. It is clearly seen that both electron–electron and electron–phonon lifetimes, which are difficult to accurately disentangle from tr-ARPES measurements standing alone, simultaneously contribute to the relaxation of excited electrons within the TSS, SR and SR* bands. In other words, the ultrafast relaxation of the linear Dirac bands forming the TSS is directly coupled to the dynamics of the spin-polarized SR and SR* states, with their appearance being triggered by the process of band inversion.

**Figure 4:**
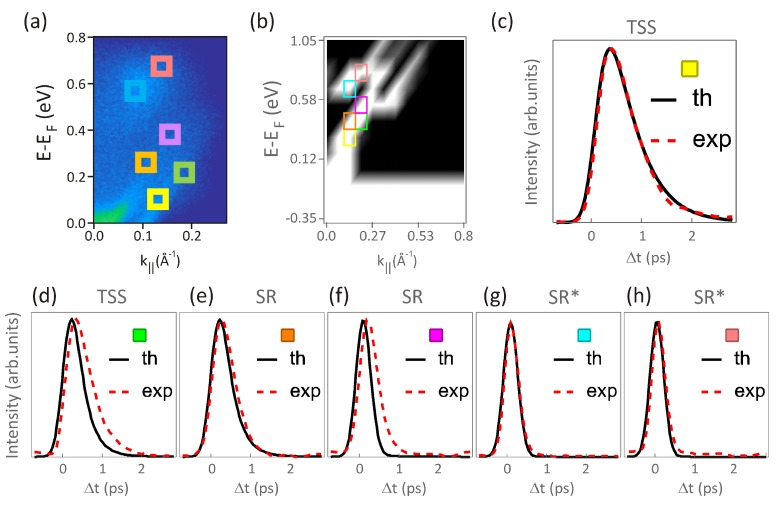
Comparison between the dynamical calculations using the GW+Boltzmann approach and the results of time-, energy-, and momentum-resolved photoemission experiments. (**a**) the band-resolved dynamics was measured by integrating the photoemission intensity within small energy-momentum windows (depicted in various colors among the different states TSS, SR and SR*) as a function of pump-probe time delay Δt; (**b**) similar analysis was performed using the discretized GW bands, with the corresponding energy-momentum windows placed at equivalent positions; (**c**–**h**) normalized intensities obtained as a function of pump-probe delay within the small energy-momentum windows shown in (**a**,**b**), respectively. Red dashed lines are the experimental spectra, and black solid lines are the corresponding calculations.

In the numerical simulations of the time evolution, a laser pulse, centred at time zero and with a time width of 180 fs, creates excitations within the system. Upon optical excitation, the most important transitions are from electronic states well below the Fermi energy to above. The highest energy states SR* decay mainly due to electron–electron scatterings into the lower energy states. This process also allows the electrons to reconstruct a Fermi–Dirac distribution at high temperature. In a slightly slower timescale, electron–phonon scatterings remove energy from the electronic system, and drive its cooling down back to room temperature. To directly compare the calculations to the experimental tr-ARPES data, we also convolute the theoretical curves with a Gaussian function of time width of 180 fs. This allows for a realistic comparison between the results of the GW+Boltzmann approach and the tr-ARPES measurements, as shown in Figure 4.

From this comparison, we find good quantitative agreement concerning both the rise times as well as the decay of the time-resolved intensities within various energy-momentum windows distributed over the different states (see Figure 4a,b). Very interestingly, even in the presence of limited time resolution, from the comparison between calculations and experiment (see Figure 4c,h), we can accurately identify the different contributions from electron–electron and electron–phonon scatterings in a quantitative way. To exemplify this, we perform a parameter-sensitive calculation by focusing on the lowest energy state within the TSS bands (yellow box in Figure 4), as this state exhibits the slower dynamics and thus it carries the fingerprint of the most important processes governing the transfer of energy from the electron to the phonon system, and subsequently to the lattice.

**Figure 5:**
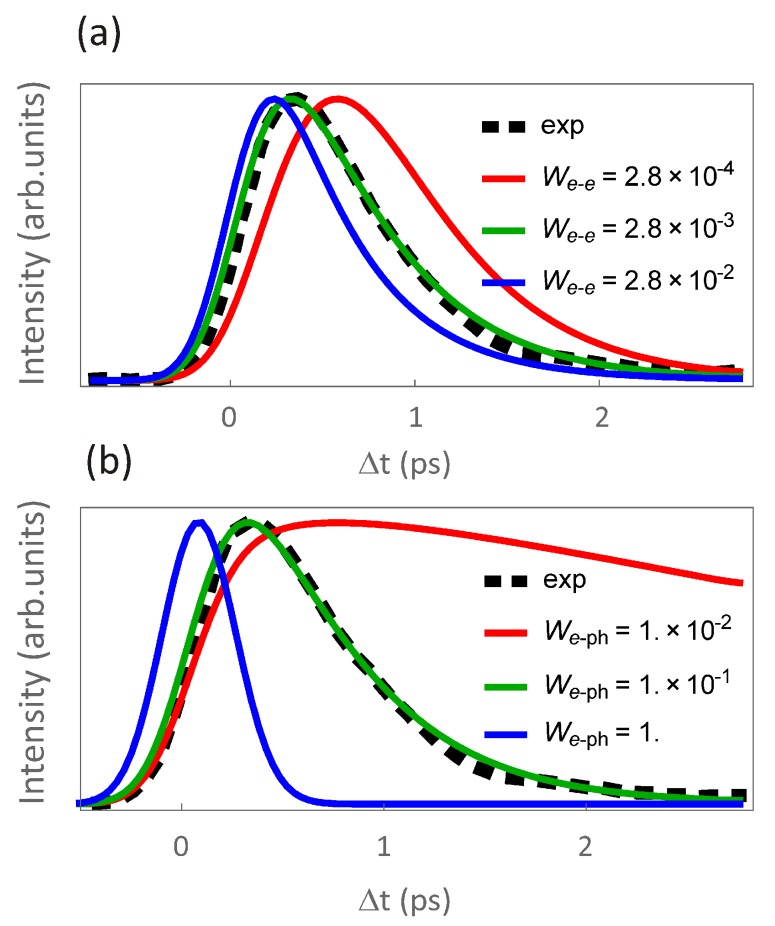
Parameter-sensitive calculation using the GW+Boltzmann approach and its comparison to experiment. (**a**) effect of changing the amplitude of the electron–electron scatterings ($W_{e-e}$) while keeping constant the strength of electron–phonon coupling ($W_{e-ph}$). Here, the time evolution of the lowest-energy state within the Dirac cone of Bi${}_{2}$Te${}_{3}$ is taken as an example; (**b**) similar calculations as in (**a**), but changing instead $W_{e-ph}$ while keeping $W_{e-e}$ constant. As indicated in the legends, black-dashed lines are the experimental result and thick solid lines of different colors the corresponding calculations.

In Figure 5a, we show the effect of changing the amplitude of the electron–electron scatterings. One clearly observes that the increasing amplitudes lead to faster scatterings in the highly-excited states, which, in turn, causes a faster feeding of electrons into the lower-energy states. This is reflected in a steeper increase of the number of electrons in the state. One also notices how the subsequent decay is not affected by electron–electron scatterings. This is readily understood if one considers that electron–phonon scatterings are more effective in reducing the energy of excited states in the vicinity of the Fermi level. The situation changes if we now compare the dynamics for different electron–phonon coupling strengths while keeping constant the amplitude of electron–electron scatterings (see Figure 5b). The longer time decay is now strongly affected, as expected. Interestingly, when the coupling is so strong that the long time decay becomes extremely fast, electron–phonon scatterings can become the dominant mechanism underlying the feeding of electrons into the TSS bands, and, therefore, the most frequent scattering process.

Finally, we would like to point out that if we would artificially set the spin polarization of SR and SR* states to zero in the calculations, the dynamics would be faster. The reason is that this would eliminate the existing constraints imposed by their spin textures, making all possible electron transitions into the Dirac cone allowed. Similarly, if we would decrease the strength of the spin–orbit interaction in the calculations, the dynamics would be faster. In contrast, if we would make the SR and SR* states artificially disappear in the calculations, the Dirac cone dynamics would be substantially slower, as there would be fewer allowed electron transitions. 

## 4. Conclusions

In conclusion, we have developed a generalized GW+Boltzmann approach for the description of ultrafast dynamics in topological insulators. This has been done by explicitly considering electron–electron and electron–phonon scatterings quantitatively, as well as the critical role of the excited spin textures and band dispersions as derived from state-of-the-art GW calculations. Taking the prototypical topological insulator Bi${}_{2}$Te${}_{3}$ as an example, we have shown good agreement between the calculations and results from time-resolved experiments, not only concerning the electronic structure but also the ultrafast response of transient electrons on the surface following femtosecond laser excitation. We have shown that the subpicosecond electron dynamics in a prototypical insulator such as Bi${}_{2}$Te${}_{3}$ is strongly affected by the existence of topologically trivial spin-polarized states accompanying the Dirac cone. We have discussed the origin of these topologically trivial states as well as their spin textures, which arise from band inversion, and, thus, from the evolution of the system, as it goes through a topological quantum-phase transition from a trivial insulator to a topological insulator. From the comparison between experiments and calculations, we have demonstrated the crucial impact that these states have on the dynamics of the topological surface state, an effect that can be understood as a widespread phenomenon in this family of materials. Finally, we have accurately obtained the magnitude and strength of electron–electron and electron–phonon couplings, and described their critical role in the electron relaxation processes taking place within individual bands.

It would be interesting to perform similar studies as the one reported here in other distinct topological phases of matter, such as type-II Weyl semimetals, where, similar to the present case, topologically trivial surface-like states accompanying non-trivial Fermi arcs have been recently discovered [60]. Concerning the theoretical approach, in future studies, it would be interesting to expand the present theory to consistently treat layer- and doping-dependent dynamics, as well as to explicitly include transport effects relevant for truly bulk-insulating phases. Our current approach could be used as a generalized theory for three-dimensional topological insulators in the bulk-conducting transport regime, paving the way for the realization of a unified theory of ultrafast dynamics in topological materials.
